# Efficient Assessment and Large-Scale Conservation of Intra-Varietal Diversity of Ancient Grapevine Varieties: Case Study Portugal

**DOI:** 10.3390/plants11151917

**Published:** 2022-07-24

**Authors:** Elsa Gonçalves, Antero Martins

**Affiliations:** 1 LEAF—Linking Landscape, Environment, Agriculture and Food Research Center, Associated Laboratory TERRA, Instituto Superior de Agronomia, Universidade de Lisboa, Tapada da Ajuda, 1349-017 Lisboa, Portugal; anteromart@isa.ulisboa.pt; 2 PORVID-Portuguese Association for Grapevine Diversity, Tapada da Ajuda, 1349-017 Lisboa, Portugal

**Keywords:** grapevine genetic resources, grapevine conservation, germplasm collection, intra-varietal variability, quantitative genetics

## Abstract

There are thousands of ancient grapevine varieties in Europe, each one having a high level of intra-varietal diversity with regard to important economic traits (yield, soluble solids content, acidity, anthocyanins, and others). However, this potential has become exposed to a process of genetic erosion since the middle of the last century. The main objective of this work is to present experimental strategies for conservation and utilization of intra-varietal diversity. A concrete example is given about the actions performed in Portugal since 1978. Two main approaches for the conservation of intra-varietal diversity were performed: (1) strict conservation (in pots and in the field without experimental design) for future generations; and (2) conservation and, simultaneously, evaluation of the intra-varietal variability for selection to fulfil the immediate needs of the grape and wine sector (in the field with experimental design). More than 30,000 accessions of Portuguese autochthonous varieties are conserved. Using the theory of mixed models, intra-varietal diversity of the yield was found for the 59 varieties studied. The conservation and the evaluation of the intra-varietal diversity for quantitative traits will allow to extract high economic value, as well as to ensure its utilization to meet the objectives of the vine and wine sector.

## 1. Introduction

Modern agriculture is generally based on plants obtained by breeding techniques. However, in viticulture, there is an almost exclusive usage of thousands of ancient varieties [1], with a high level of inter-varietal diversity. This diversity is an important factor of adaptation to multiple growing environments and sustains the quality, identity, and differentiation of grapes, wines, and other derived products.

Field conservation, in vitro, and cryopreservation are frequently referred tools for conservation of *Vitis* genetic resources [2], which main concerns are usually focused on inter-varietal diversity. The traditional and widely used field conservation in ampelographic collections of inter-varietal diversity is well established. A reference example is the largest collection of grapevine varieties maintained in Vassal (France), but there are also national and regional ampelographic collections maintained all over the world. However, the conservation of one or few clones of an ancient variety is far from guaranteeing its true conservation for the purpose of adapting to future changes and the sustainability of viticulture. In fact, a high level of diversity of quantitative traits exists within each variety, known as the intra-varietal diversity, which include those of greatest economic importance (yield, soluble solids content, acidity, anthocyanins and many others as abiotic and biotic stress tolerances). This heterogeneity ensures stable behavior of the variety in different environments (low genotype × environment interaction) and constitutes the raw material for carrying out the selection within the variety with high genetic and economic gains.

As different accessions of a variety have a unique ampelographic description, intra-varietal diversity is sometimes not well understood and valued, but it is a reality in fact. At a given time after domestication, a plant started to be vegetatively multiplied and variability has gradually been created over the centuries through the accumulation of somatic mutations and other DNA instability mechanisms. Molecular understanding and detection of intra-varietal diversity has been the subject of many studies [3,4,5,6,7,8,9]. However, the purposes of conserving intra-varietal diversity for the future and exploiting it for satisfying the immediate needs of the grape and wine sector have been insufficiently considered in current approaches applied to grapevine worldwide. Nonetheless, concern with intra-varietal diversity has been reported in this last perspective, for example, in France [10,11,12,13], Germany [14], Italy [15,16], Spain [17], Switzerland [18,19], and Portugal. In the latter country, a methodology for conservation and evaluation of intra-varietal diversity and selection within a variety has been developed since 1978 [20,21,22,23].

Unfortunately, the intra-varietal diversity of ancient varieties has become exposed to a new and serious risk due to the emergence of a rapid and dramatic process of genetic erosion in the middle of the last century. In fact, the abandonment of the technique of grafting in the field (with buds of diverse origins) in favor of planting bench-grafted plants (made with buds from a few homogeneous plots dedicated to this purpose) and the generalization of the selection with a narrow genetic basis (focused on the homogeneous clone) have been damaging inter and intra-varietal diversity. This concern was recognized by the International Organization of Vine and Wine in the resolution OIV-VITI 424/2010 [24], “Conservation of Vine’s Genetic Resources” and in the Annex II of the resolution OIV-VITI 564B-2019 [25], “OIV Process for the recovery and conservation of the intra-varietal diversity and the polyclonal selection in grape varieties with wide genetic variability”. Additionally, the major advances in knowledge of grapevine viruses have accentuated the focus of selection based on diagnosis and elimination of certain viruses, with the secondary consequence of narrowing that genetic diversity [26]. As a result, the vineyards are no longer planted with the whole variety, but only with a small part of it (at the limit, a clone), leading to the fixation of the variety with the interesting traits at present, but preventing its adaptation to new objectives in the future. Stopping this process of genetic erosion is urgent since diversity is a natural feature of Europe and is still concentrated in its older vineyards. Therefore, the conservation of intra-varietal genetic variability became a crucial strategy to preserve traditional viticulture and to face future challenges (climate change, biotic and abiotic stresses, consumer demands, etc.).

The main objective of this work is precisely to outline experimental strategies for the conservation and utilization of intra-varietal diversity, consolidating a competitive advantage provided by the originality and history of ancient grapevine varieties and contributing to the promotion of sustainable development of the vine and wine sector.

The scheme of the methodology of intra-varietal variability conservation, evaluation, and selection within an ancient grapevine variety was recently updated by [23]. According to these authors, a well-conducted conservation of intra-varietal diversity for an ancient variety involves a previous prospection of plants in the old vineyards to obtain a representative sample of the variety in its main growing regions. Those plants are then collected, multiplied, and planted or grafted to install a collection of intra-varietal diversity. Two main approaches for the conservation of intra-varietal diversity were pointed out [23]: (1) strict conservation and (2) conservation and, simultaneously, evaluation of the intra-varietal variability for selection to fulfil the immediate needs of the grape and wine sector. The first approach (strict conservation) is applied to all autochthonous varieties. The conservation is performed in pots (usually four plants per genotype) or in the field with a single plot per genotype (usually six plants per genotype). The objective is to save the intra-varietal diversity to allow its use for future generations. The second approach (conservation and evaluation) is applied to grapevine varieties with particular importance and widely used at present. In this case, the evaluation of important quantitative traits (such as yield, must quality traits, and tolerance to abiotic stresses) and polyclonal selection is desirable. For this purpose, a field trial is established with hundreds of genotypes representative of the intra-varietal diversity, with repetitions (usually four to six), and an appropriate experimental design (randomized complete block designs or designs of the family of incomplete block designs, such as alpha and row-column designs) [23,27]. As a consequence, the field trial for the evaluation of intra-varietal diversity for economically important traits and for polyclonal selection also constitutes a conservation strategy, valid for at least 30 years.

This latter approach is the methodology of polyclonal selection described in the Resolution OIV-VITI 564B-2019 [25] of the International Organization of Vine and Wine “OIV Process for the recovery and conservation of the intra-varietal diversity and the polyclonal selection in grape varieties with wide genetic variability”.

The objectives of this work are: (1) to demonstrate the implementation of the methodology of conservation of intra-varietal diversity in Portugal; (2) to validate the utility of the conservation in field trials to respond to the immediate needs of the vine and wine sector.

## 2. Results

### 2.1. Conservation of Intra-Varietal Diversity

At present, the results concerning the conservation of intra-varietal diversity in Portugal are presented in Table 1: 14,969 accessions conserved in pots of at least 218 identified varieties (the exact number depends on ongoing molecular testing for doubtful cases), each accession represented by 4 plants; 4308 accessions in field conservation without experimental design, each accession represented by one plot with 6 plants; and 10,938 accessions in field trials for evaluation of intra-varietal diversity and polyclonal selection. For this latter conservation, the variety, the number of accessions for each variety, and the type of experimental design for a big part of the field trials planted since 1978 in Portugal are described in Table 2.

For the different varieties, the number of accessions (clones) is variable, according to their importance, presence in old vineyards, and dispersion in growing regions. The value ranges from less than 10 accessions (for varieties that at present are scarcely found in the old vineyards and for which the prospection is ongoing) to more than 450 accessions (for varieties that are important and widely grown). For example, by taking the sum of all different accessions in field and pots, 676 clones of Castelão are conserved, as well as 583 of Rufete, 571 of Alvarinho, 566 of Arinto, 556 of Baga, 543 of Trincadeira, 499 of Síria, 493 of Bastardo, 465 of Touriga Nacional, and 467 of Fernão Pires.

In total, 30,168 accessions are already conserved. Among these accessions, there is a considerable number of redundant collections in field trials with fully replicated designs for clonal selection (mainly dedicated to the study of genotype × environment interaction in 30–40 clones of 45 varieties) and in other field trials installed for methodological studies.

### 2.2. Evaluation of the Intra-Varietal Diversity of Yield

The evaluation of the intra-varietal diversity of yield was performed for 59 varieties, whose experimental designs are described in Table 2. The results for the several quantitative genetic parameters are shown in Table 3. Whenever possible, data analysis was based on the average yield values observed over several years.

For all studied varieties, the applied methodology allowed the identification of significant intra-varietal diversity for yield (rejection of $H_{0}:\sigma_{g}^{2}=0$, *p*-value < 0.05), although the precision associated to the estimate of genotypic variance (value for the ratio ${\hat{\sigma }}_{g}^{2}$/SE) depended on the field trial. The values of broad-sense heritability ($H^{2}$) are provided to assess the part of the variance of phenotypic yield data explained by genotypic causes. The values obtained for $H^{2}$ ranged from 0.261 to 0.956. However, it is worth emphasizing that for 54 varieties, the value of $H^{2}$ was higher than 0.54, and for 23 varieties, it was higher than 0.70. For several varieties/field trials, both higher values of $H^{2}$ and higher precision associated to genotypic variance component (higher value ${\hat{\sigma }}_{g}^{2}$/SE) were found (for example, Arinto, Touriga Nacional, Tinta Caiada, Rabo de Ovelha, Síria, Viosinho, Vital, Castelão, Antão Vaz, and Gouveio varieties).

A relative measure of intra-varietal diversity for yield is given by the coefficient of genotypic variation (${CV}_{G}$), which provides an insight into the diversity within the variety. Among the varieties studied, it varied from 7.3% to 43.0%. Another type of information directly related to the existing intra-varietal diversity is the range of variation of a relative measure of the Empirical Best Linear Unbiased Predictors (EBLUPs) of genotypic effects of the yield (the EBLUPs expressed in percentage of the population mean). The part of the difference between the phenotypic mean of the genotype and the overall mean of the population, which is explained by genetic causes, is the EBLUP of genotypic effect. The amplitude of variation between the lowest and the highest EBLUP of genotypic effects of the yield was in general lower for varieties with lower values of $H^{2}$, ${CV}_{G}$, and ${\hat{\sigma }}_{g}^{2}$/SE (such as Moreto, Avesso, Touriga Franca, Alfrocheiro, and Fonte Cal varieties). In contrast, the higher variation in EBLUPs of the genotypic effects of yield was observed for varieties where higher values of $H^{2}$, $CV_{G}$, and ${\hat{\sigma }}_{g}^{2}$/SE were found (such as the Sercial, Negra Mole, Tinta Miúda, Vital, Tinta Caiada, Rabo de Ovelha, Síria, Arinto, Touriga Nacional, Viosinho, and Castelão varieties). For example, for the Arinto variety, the predicted genotypic yield varied from 61.59% lower than the mean of the population to 58.32% higher than the mean of the population and for Touriga Nacional from 57.81% lower to 51.76% higher than the mean of the population. Even in the cases where less intra-varietal diversity was observed, the range between the lowest and the highest EBLUP deserves attention. For example, for the Moreto variety, the predicted genotypic yield of the genotype with best EBLUP is 11.3% higher than the mean of the population and the predicted genotypic yield of the worst genotype is 9.7% lower than the mean of the population. This finding is an indicator of the possible genetic gains than can be obtained with selection and demonstrates the practical utility of conserving and evaluating intra-varietal diversity.

## 3. Discussion

Few references are found on the strategies of conservation of intra-varietal diversity opposite to what would be desirable given the importance of the subject. However, the status report of this type of conservation in France was provided by [12,13], in Spain (Rioja) by [17], in Italy by [16], and in Germany by [14]. In Portugal, a planned and large-scale approach has been adopted for the conservation of intra-varietal diversity, consisting of the conservation of representative samples of genotypes of all the autochthonous Portuguese grapevine varieties in pots and/or in the field. The strategic nature and long-term projection of conservation led to the creation of a dedicated farm for this purpose: the Experimental Centre for the Conservation of Grapevine Diversity of the Portuguese Association for Grapevine Diversity (PORVID). In 4 years’ time, a total of 50,000 accessions of all Portuguese autochthonous varieties are expected to be conserved.

As mentioned before, the different types of conservation of intra-varietal diversity presented in this work have different objectives. When conservation is performed in pots or in the field with a single plot per accession its only purpose is strict conservation. The only evaluation that can be obtained is the mean phenotypic value of the experimental unit for a target trait. Therefore, without replications, there is no way to assess the error and genotypic variance estimates nor to predict the genotypic effects [28,29]. Hence, there is no basis for any useful quantification of intra-varietal diversity and selection of quantitative traits [21,23]. When the conservation is performed in field trials following the basic rules of experimental design (independent repetitions and appropriate randomization), the separation of phenotypic variability into its different sources of variation is performed. Consequently, the quantification of intra-varietal diversity of important quantitative traits (such as yield and must quality traits) and selection of a superior group of clones (polyclonal selection) with prediction of genetic gains are feasible. In this work, this latter approach was applied to yield in 59 varieties (Table 3).

Unfortunately, the analyses of quantitative genetics performed in Table 3 are not common in the context of grapevine, in contrast to what is currently done for other crops [30,31]. The importance of having field trials designed as those described in Table 2 should be emphasized. In fact, they are the starting point for any efficient selection of quantitative traits considering either classic quantitative genetics or genomics.

The results obtained for the 59 varieties should be seen individually, as quantitative genetic parameters are specific to each population, field trial, and year. The results obtained for all varieties proved the suitability of these field experiments to quantify intra-varietal diversity and perform selection.

The values for $H^{2}$ were in general high and resulted from the ability of the experimental design to control the background noise of the field and from the genotypic variability within each variety (the higher the capacity of the experimental design to control the background noise and/or the higher the genotypic variability within a variety, the higher the value of $H^{2}$). The precision of the estimate of genotypic variance depends on the number of accessions that constitutes the representative sample being evaluated (the higher the number of accessions in the field trial, the higher the precision associated to the estimate of the genotypic variance component). In practice, this finding can be confirmed by crossing the data on the varieties presented in Table 2 and Table 3, where the number of evaluated accessions and the precision of ${\hat{\sigma }}_{g}^{2}$ are provided, respectively. 

The origin of intra-varietal diversity has already been justified by several molecular mechanisms of variation [3,4,5,6,7,8,9]. In this work, genetic differences in yield were found within all the 59 varieties studied, separating the different sources of variation of yield phenotypic data through the fitting of a linear mixed model and using quantitative genetic tools. The intra-varietal diversity is a function of time since the domestication, but no less important, it is a function of the intensity of cultivation of the variety throughout history (which determines the number of multiplications). For example, this latter factor justifies the results of intra-varietal diversity found in Seara Nova and Dona Maria (cultivars obtained by artificial crossing in Portugal in the middle of the last century). In contrast, it justifies the low level of intra-varietal diversity observed in Moreto, a variety from a very restricted region of Alentejo, which has been very little cultivated. On the other hand, for important widely grown varieties in Portugal, such as Arinto, Sercial, Touriga Nacional, Rabo de Ovelha, Malvasia Fina, and Negra Mole, a large amount of intra-varietal diversity with high precision in the estimate of genotypic variance component were found in the field trials evaluated.

It is unfeasible to discuss the results of all the 59 varieties in this study. However, the results regarding intra-varietal diversity are of great interest as they highlight the cultural context of grapevine varieties and provide new rational guidance for selection and conservation. In this work, the immediate utility of intra-varietal diversity is supported by the wide range of variation observed for the EBLUPs of the genotypic effects of the yield, which clearly shows that there is raw material to perform selection with high genetic gains. In fact, notable levels of variation within a variety have been reported. For example, for Portuguese autochthones varieties, variations of more than ten-fold in yield or two-fold in soluble solids were registered [20,21]; for Pinot noir, large variations in yield and acidity were also found [32]. The utility of intra-varietal diversity of some varieties has also been demonstrated for other important traits [33], including for abiotic stress tolerance [34,35,36].

In short, the greater the intra-varietal diversity conserved and evaluated, the greater the genetic gains obtainable from the selection and the capacity of a variety to respond to present and future challenges of viticulture. Therefore, the purposes of conserving intra-varietal diversity for the future should be highly considered in grapevine worldwide.

## 4. Materials and Methods

### 4.1. Conservation of Intra-Varietal Diversity

To efficiently conserve intra-varietal genetic variability of a variety, the key point is the process to obtain a representative sample of that diversity. This process is described in detail in [21,23,25].

The accessions conserved were prospected in vineyards pre-dating the development of clonal selection programs and bench grafting nursery activities in Portugal (before 1980), which are those that preserve the diversity that was created in the past. For each variety, the prospection was conducted in its main ancient growing regions. In each region, prospection of geographically distant and unrelated vineyards was performed (from different owners, different years of planting, etc.). The total number of plants came from the largest possible number of vineyards (20 or more per region), and only a few plants from each vineyard were sampled (5 or less). Within each vineyard, the plants were marked randomly (except in cases of serious diseases of a systemic type). When possible, the objective was to obtain a minimum of 70 accessions per region and per variety. The plants marked were subjected to diagnosis of highly frequent viruses, collected, multiplied, and planted or grafted to install a germplasm collection.

Since 1978, the prospection of the intra-varietal diversity of important varieties was concentrated in one or two years, followed by its conservation in the field, mostly in field trials. Since 2011, accessions of all Portuguese autochthonous varieties have been conserved every year in pots (four plants per accession). The objective is to assemble the prospected accessions to obtain a representative sample of the intra-varietal diversity of each variety of all the autochthonous varieties.

### 4.2. Evaluation of Intra-Varietal Diversity of the Yield

For the evaluation of intra-varietal diversity, data were collected in field trials of 59 varieties (those described and identified in bold in Table 2). The experimental designs of those field trials have been generated in CycDesigN software (http//www.vsni.co.uk/software/cycdesign/ (accessed on 16 May 2022)) since 2005.

Several traits are usually measured in those field trials, but the one under consideration to exemplify the methodology proposed in this paper was the yield. This quantitative trait is the most feasible to measure (it only involves the weight in the field); therefore, it is the one with more data available for more varieties and years. Additionally, in previous studies, it was also the trait that showed widest range of variation [32,33].

Yield data from several years were usually considered for all the varieties studied. In each year, this trait was evaluated in all replicates of the field trial. The grapes of all plants in each experimental unit of each accession were weighted, and in the statistical analysis, the mean yield of each experimental unit (kg/plant) was used for each repetition of each accession.

For the quantification of the intra-varietal diversity, the statistical analysis is focused on the estimation of the genotypic variance component. In a field trial, the estimate of the genotypic variance component obtained for the yield will differ among years. According to [23], this occurs for several reasons: (1) data scales differ among years; (2) several sources of error variation are present (such as sampling, evaluation errors, and other environmental deviations among years); and (3) the range of genetic differences among accessions differs with the year due to G × E interaction. Therefore, for an overall view and a more precise quantification of the genetic diversity within a variety, the average of several years should be used [23]. Hence, in the statistical analysis of the present study, the average of several years was used for each repetition of each accession whenever possible according to the experimental designs described in Table 2.

Yield data analysis was based on linear mixed models. The theory of mixed models has been applied to a wide scope of sciences [28,37] and it is the basis for the analysis of intra-varietal diversity of yield and other quantitative traits in grapevine field trials. Examples of mixed models that are applied to data from grapevine selection field trials are described in [27,38,39,40,41].

In the models used in this work, since a random sample of accessions (clones) of the variety was studied, the genotypic effects were considered to be random. The effects of the experimental design were also assumed as random (the effects of complete blocks, in the case of randomized complete block design; the effects of resolvable replicates and the effects of rows and columns nested within resolvable replicates, in the case of resolvable row-column design). Random errors and all random effects were assumed to be independent and identically distributed normal random variables. All random effects were assumed to be mutually independent. The residual maximum likelihood (REML) estimation method was used for the estimation of the covariance parameters [42]. The linear mixed models were fitted using ASReml-R package [43] within R [44].

For each variety, the intra-varietal diversity among the evaluated accessions ($H_{0}:\sigma_{g}^{2}=0\;\mathrm{vs}.\;H_{1}:\sigma_{g}^{2}>0$) was tested using a residual maximum likelihood ratio test. As the null hypothesis was on the boundary of the parameter space, the *p*-value of the test was assumed to be half of the reported *p*-value from the chi-squared distribution with one degree of freedom [45,46]. For the evaluation of the precision of the quantification of the intra-varietal diversity, the ratio ${\hat{\sigma }}_{g}^{2}$/SE was considered (where ${\hat{\sigma }}_{g}^{2}$ is the estimate of the genotypic variance of the yield and SE is the standard error associated to this estimate). The higher the value for this ratio, the higher the precision associated to the quantification of intra-varietal diversity.

Additionally, for each variety, the following genetic indicators were computed: (1) a generalized measure of broad-sense heritability ($H^{2}$) [39], to evaluate how much phenotypic variability is due to genetic causes and to judge the efficiency of selection; (2) a relative measure of intra-varietal genetic diversity—the genotypic coefficient of variation, in percentage—to obtain a clearer interpretation of the results of the intra-varietal diversity ($CV_{G}$, the estimate of the genotypic standard deviation of the yield divided by the yield mean of the population); and (3) through the mixed model equations, the Empirical Best Linear Unbiased Predictors (EBLUPs) of the genotypic effects of the yield were obtained [28,47]. The part of the difference between the phenotypic mean of the accession and the overall mean of the population, which is explained by genetic causes, is the EBLUP of genotypic effect. The range of variation between the lowest and the highest values of EBLUPs (expressed as the percentage of the mean of the population) was used to evaluate the range of intra-varietal genetic diversity and the potential gains of selection.

## 5. Conclusions

This paper demonstrated the implementation of a methodology of the conservation of intra-varietal diversity in Portugal. A coherent approach for conserving and exploring intra-varietal diversity of ancient grapevine varieties is currently implemented in Portugal by PORVID. This work is supported by a network of field trials planted all over the country and by PORVID’S Experimental Centre for the Conservation of Grapevine Diversity. At present, more than 30,000 accessions of at least 218 varieties are conserved.

The validation of the utility of the conservation in field trials to respond to the immediate needs of the vine and wine sector was performed by the recognition of intra-varietal diversity in yield for the 59 varieties studied and was also supported by the wide range of variation observed in the EBLUPs of the genotypic effects of the yield, which clearly shows that there is raw material to perform selection with high genetic gains.

The conservation and the exploitation of intra-varietal diversity are crucial to the knowledge of the origin and evolution of ancient varieties, the selection and achievement of high genetic gains in yield and must quality traits, the adaptation to biotic and abiotic factors, and the prevention of genetic erosion. That is, the conservation and the exploitation of intra-varietal diversity is essential to the sustainability of the vine and wine sector.

## Figures and Tables

**Table 1:** Types of conservation of the intra-varietal diversity of varieties in Portugal.

Type of Conservation	No. Varieties	No. Accessions
Pots	218	14,969
Field	31	4308
Field trials	64	10,938

**Table 2:** Conservation in fully replicated field trials of 63 varieties in Portugal for evaluation of intra-varietal diversity and polyclonal selection (CRD, complete randomized design; RCBD, randomized complete block design; RCD, resolvable row-column design; Alpha, Alpha design; ν, number of genotypes; r, number of complete blocks; k, number of rows nested within complete block; s, number of columns nested within a complete block; p, plants per experimental unit (plot)). The yield data collected in the field trials highlighted in bold were used to evaluate the intra-varietal diversity in this study. For varieties with more than one field trial identified with *, different accessions were conserved. The named regions are wine regions; when the country is not mentioned, the regions are from Portugal.

Variety	Prospected Regions	No. Accessions	Experimental Design
**Alfrocheiro**	Dão	237	RCBD: r=5, p=3
**Alvarelhão**	Dão, Douro	32	RCBD: r=4, p=6
Alvarinho *	Vinhos Verdes	196	RCBD: r=3, p=3
Alvarinho *	Vinhos Verdes, Rías Bajas (Spain)	180	RCD: r=2, k=12, s=15, p=3; Alpha: r=2, k=6, s=30, p=3
**Amaral**	Vinhos Verdes, Lafões	137	RCBD: r=2, p=3
**Antão Vaz**	Alentejo *	210	RCBD: r=5, p=5
Antão Vaz	Alentejo *	110	RCD: r=6, k=11, s=10, p=3
**Aragonez**	Alentejo, Douro, Rioja (Spain), Valdepeñas (Spain)	257	RCBD: r=5, p=3
Arinto *	Vinhos Verdes, Bairrada, Lafões, Lisboa	247	RCBD: r=5, p=4
Arinto *	Vinhos Verdes, Bairrada, Lafões, Lisboa	165	RCD: r=6, k=11, s=15, p=3
**Arinto dos Açores**	Açores	77	Alpha: r=3, k=6, s=13, p=4
**Avesso**	Vinhos Verdes	164	RCBD: r=4, p=4
**Azal**	Vinhos Verdes	219	RCBD: r=4, p=4
**Baga**	Bairrada, Dão	200	CRD: r=5, p=4
Bastardo	Dão, Beira Interior, Douro, Trás-os-Montes, Jura (France)	272	RCBD: r=4, p=3
**Bastardo**	Dão, Beira Interior, Douro, Trás-os-Montes, Jura (France)	374	RCD: r=4, k=17, s=22, p=3
**Batoca**	Beira Interior, Vinhos Verdes	92	RCD: r=5, k=5, s=19, p=5
**Bical**	Bairrada, Dão	240	RCBD: r=5, p=3
**Borraçal**	Vinhos Verdes	200	RCBD: r=3, p=3
**Camarate**	Bairrada, Beira Interior, Dão, Lisboa	242	RCBD: r=4, p=4
**Castelão**	Alentejo, Lisboa, Península de Setúbal, Tejo	270	RCD: r=5, k=18, s=15, p=3
Castelão	Alentejo, Lisboa, Península de Setúbal, Tejo	209	RCD: r=6, k=11, s=19, p=3
**Cerceal Branco**	Dão, Douro	50	RCBD: r=5, p=3
**Cercial**	Bairrada	50	RCBD: r=4, p=3
Códega do Larinho	Douro, Trás-os-Montes	78	RCBD: r=6, p=3
**Dona Maria**	Alentejo, Algarve, Lisboa	85	RCBD: r=4, p=7
**Encruzado**	Dão	180	RCD: r=4, k=10, s=18, p=3
Encruzado	Dão	179	RCBD: r=2, p=3
**Espadeiro**	Vinhos Verdes	133	RCBD: r=5, p=2
**Fernão Pires**	Bairrada, Dão, Lisboa, Tejo	232	CRD: r=3, p=4
**Fonte Cal**	Beira Interior	220	RCBD: r=5, p=3
Gouveio *	Dão, Douro, Trás-os-Montes	143	RCBD: r=4, p=4
Gouveio *	Dão, Douro, Trás-os-Montes	154	RCD: r=5, k=14, s=11, p=3
**Grenache**	Cariñena (Spain), Mentrida (Spain), Vaucluse (France), Sardegna (Italy)	126	RCD: r=6, k=6, s=21, p=3
Grenache	Cariñena (Spain), Mentrida (Spain), Vaucluse (France), Sardegna (Italy)	215	RCBD: r=5, p=3
Jaen *	Dão	200	RCBD: r=6, p=4
Jaen *	Dão, Bierzo (Spain)	206	RCBD: r=5, p=4
**Jampal**	Lisboa	180	RCBD: r=4, p=4
**Loureiro**	Vinhos Verdes	250	RCBD: r=5, p=2
**Malvasia**	Lisboa	27	RCBD: r=4, p=3
**Malvasia Fina**	Dão, Douro, Lisboa	180	RCBD: *r* = 5, *p* = 3
**Moreto**	Alentejo	66	RCD: *r* = 6, *k* = 11, *s* = 6, *p* = 3
**Moscatel Galego Branco**	Douro	200	RCBD: *r* = 5, *p* = 5
**Moscatel Galego Roxo**	Douro, Península de Setúbal	13	RCBD: *r* = 5, *p* = 3
Moscatel Graúdo *	Península de Setúbal	187	RCBD: r=5, p=4
Moscatel Graúdo *	Península de Setúbal	99	RCBD: r=4, p=4
**Negra Mole**	Algarve	196	RCBD: r=5, p=4
Padeiro	Vinhos Verdes	102	RCBD: r=3, p=3
**Rabigato**	Douro	127	RCBD: r=5, p=3
**Rabo de Ovelha**	Alentejo, Dão, Douro, Lisboa	250	RCBD: r=4, p=5
**Ramisco**	Lisboa	59	RCBD: r=4, p=3
**Ratinho**	Lisboa	194	RCBD: r=5, p=4
**Rufete**	Beira Interior, Dão, Douro	242	RCD: r=6, k=11, s=22, p=3
**Seara Nova**	Lisboa	40	RCBD: r=5, p=7
**Sercial**	Douro, Lisboa, Madeira	148	RCBD: r=5, p=3
**Síria**	Algarve, Alentejo, Dão, Douro, Beira-Interior	239	RCBD: r=5, p=4
**Terrantez do Pico**	Açores	70	RCBD: r=3, p=4
Tinta Barroca *	Douro	190	RCBD: r=5, p=4
Tinta Barroca *	Douro	210	RCBD: r=4, p=3
Tinta Caiada	Alentejo, Douro, Somontano (Spain)	208	RCD: r=5, k=13, s=20, p=3
**Tinta Caiada**	Alentejo, Douro, Somontano (Spain)	220	RCD: r=6, k=11, s=16, p=3
**Tinta Francisca**	Douro	61	RCBD: r=3, p=4
**Tinta Miúda**	Lisboa	100	RCBD: r=4, p=4
**Tinta Negra**	Lisboa	19	RCBD: r=4, p=3
**Tinto Cão**	Douro	168	RCBD: r=5, p=4
Touriga Fêmea	Dão, Douro	70	RCD: r=5, k=7, s=10, p=3
Touriga Franca *	Douro	90	RCBD: r=4, p=8
Touriga Franca *	Douro	110	RCBD: r=5, p=3
Touriga Franca *	Douro	98	RCD: r=5, k=7, s=14, p=3
Touriga Nacional *	Dão, Douro	69	RCBD: r=3, p=8
Touriga Nacional *	Dão, Douro	197	RCBD: r=5, p=4
Touriga Nacional	Dão, Douro	189	RCD: r=5, k=7, s=27, p=3
**Trajadura**	Vinhos Verdes	237	CRD: r=4, p=2
**Trincadeira**	Alentejo, Beira-Interior, Dão, Douro, Lisboa, Tejo	271	RCBD: r=5, p=4
**Uva Cão**	Dão	77	RCD: r=6, k=11, s=7, p=3
**Verdelho**	Açores	72	RCD: r=3, k=8, s=9, p=4
**Vinhão**	Vinhos Verdes	211	RCBD: r=5, p=2
**Viosinho**	Douro	203	RCBD: r=5, p=3
**Vital**	Douro, Lisboa, Tejo	232	RCBD: r=4, p=4

**Table 3:** Quantification of intra-varietal diversity of the yield in 59 varieties in Portugal (overall yield mean of the field trial (kg/plant); ${\hat{\sigma }}_{g}^{2}$ is the estimate of the genotypic variance of the yield and SE is the standard error associated to this estimate; the ratio ${\hat{\sigma }}_{g}^{2}$/SE; broad-sense heritability ($H^{2}$); genotypic coefficient of variation, in percentage ($CV_{G}$); minimum and maximum empirical best linear unbiased predictors (EBLUPs) of the genotypic effects expressed as the percentage of the mean of the population (MinEBLUP and MaxEBLUP, respectively)).

Variety (Years of Evaluation)	Overall Mean	${\hat{\sigma }}_{g}^{2}\;(\mathrm{SE})$	${\hat{\sigma }}_{g}^{2}/\mathrm{SE}$	$H^{2}$	$CV_{G}\;\left(\%\right)$	Min EBLUP (%)	Max EBLUP (%)
Alfrocheiro (one year)	1.634	0.053 (0.018)	2.846	0.272	14.0	−17.10	23.03
Alvarelhão (average of 4 years)	2.015	0.142 (0.047)	3.001	0.766	18.7	−31.57	37.52
Alvarinho (average of 3 years)	2.569	0.361 (0.052)	6.940	0.716	23.4	−47.70	60.79
Amaral (average of 8 years)	4.190	0.816 (0.161)	5.053	0.656	21.6	−45.60	38.99
Antão Vaz (average of 3 years)	2.945	0.378 (0.048)	7.894	0.777	20.9	−48.50	48.69
Aragonez (average of 5 years)	2.585	0.235 (0.035)	6.793	0.611	18.8	−38.34	35.86
Arinto (average of 4 years)	1.390	0.123 (0.014)	8.839	0.802	25.3	−61.59	58.32
Arinto dos Açores (average of 2 years)	0.470	0.006 (0.003)	1.943	0.331	16.9	−23.11	33.99
Avesso (average of 4 years)	3.881	0.140 (0.062)	2.274	0.261	9.6	−12.63	11.79
Azal (average of 3 years)	4.960	0.591 (0.150)	3.942	0.405	15.5	−28.22	28.23
Baga (average of 4 years)	2.210	0.097 (0.017)	5.543	0.574	14.1	−26.97	36.01
Bastardo (average of 4 years)	4.607	0.604 (0.069)	8.769	0.653	16.9	−58.77	38.89
Batoca (one year)	2.180	0.347 (0.083)	4.165	0.635	27.0	−47.36	78.32
Bical (average of 5 years)	2.542	0.100 (0.016)	6.414	0.598	12.5	−30.37	22.75
Borraçal (average of 4 years)	5.768	0.733 (0.167)	4.387	0.469	14.8	−34.37	28.97
Camarate (average of 4 years)	1.513	0.056 (0.008)	7.121	0.660	15.6	−48.17	40.89
Castelão (average of 7 years)	5.871	1.450 (0.182)	7.950	0.706	20.5	−57.07	43.42
Cerceal-Branco (average of 5 years)	3.061	0.192 (0.059)	3.240	0.660	14.3	−31.06	21.46
Cercial (average of 7 years)	2.121	0.104 (0.030)	3.501	0.714	15.2	−31.78	26.90
Dona Maria (one year)	6.716	1.295 (0.248)	5.216	0.819	16.9	−41.88	27.19
Encruzado (average of 8 years)	2.927	0.272 (0.042)	6.449	0.693	17.8	−41.31	29.10
Espadeiro (average of 5 years)	8.412	2.999 (0.513)	5.846	0.731	20.6	−39.35	36.12
Fernão Pires (average of 3 years)	1.889	0.096 (0.018)	5.327	0.523	16.4	−30.68	33.02
Fonte Cal (average of 4 years)	2.618	0.104 (0.026)	3.998	0.402	12.3	−17.62	25.59
Gouveio (average of 2 years)	3.731	0.693 (0.098)	7.083	0.824	22.3	−41.61	49.62
Grenache (average of 2 years)	5.870	1.253 (0.216)	5.800	0.738	19.1	−51.55	37.05
Jaen (average of 2 years)	1.587	0.111 (0.021)	5.293	0.536	21.0	−32.73	42.21
Jampal (average of 3 years)	1.643	0.102 (0.021)	4.864	0.545	19.5	−40.62	37.74
Loureiro (average of 2 years)	2.478	0.228 (0.056)	4.042	0.515	19.3	−37.27	38.49
Malvasia (average of 9 years)	2.655	0.401 (0.137)	2.936	0.831	23.9	−37.00	39.48
Malvasia Fina (average of 2 years)	0.990	0.100 (0.023)	4.320	0.469	31.9	−39.80	71.80
Moreto (average of 2 years)	6.407	0.221 (0.102)	2.175	0.416	7.3	−9.70	11.28
Moscatel Galego ((average of 4 years)	2.380	0.281 (0.041)	6.818	0.691	22.3	−43.38	40.28
Moscatel Graúdo (average of 3 years)	2.121	0.202 (0.033)	6.164	0.647	21.2	−48.94	42.96
Negra Mole (average of 2 years)	0.919	0.115 (0.019)	6.101	0.643	36.9	−56.61	104.91
Rabigato (average of 2 years)	1.485	0.209 (0.041)	5.072	0.647	30.8	−53.87	75.34
Rabo de Ovelha (average of 3 years)	1.653	0.286 (0.033)	8.583	0.775	32.3	−61.49	61.78
Ramisco (average of 8 years)	1.785	0.118 (0.052)	2.259	0.456	19.2	−35.95	25.25
Ratinho (average of 2 years)	4.116	0.379 (0.065)	5.827	0.603	14.9	−38.95	27.89
Rufete (one year)	3.723	0.224 (0.034)	6.490	0.600	12.7	−36.83	23.99
Seara Nova (one year)	2.690	0.134 (0.059)	2.262	0.516	13.6	−24.48	23.18
Sercial (average of 2 years)	1.194	0.264 (0.039)	6.691	0.792	43.0	−58.85	132.46
Síria (average of 2 years)	1.955	0.231 (0.027)	8.431	0.777	24.6	−77.53	43.99
Terrantez do Pico (average of 4 years)	1.506	0.256 (0.056)	4.590	0.878	33.6	−75.17	50.05
Tinta Barroca (average of 3 years)	1.803	0.112 (0.023)	4.936	0.487	18.6	−30.77	43.58
Tinta Caiada (average of 2 years)	5.877	2.647 (0.307)	8.632	0.826	27.7	−62.12	68.76
Tinta Francisca (one year)	0.654	0.048 (0.015)	3.149	0.593	33.6	−51.49	57.68
Tinta Miúda (average of 8 years)	2.436	0.537 (0.080)	6.724	0.956	30.1	−92.93	53.12
Tinta Negra (average of 8 years)	1.797	0.411 (0.196)	2.099	0.855	35.7	−48.85	56.29
Tinto Cão (average of 3 years)	1.277	0.039 (0.007)	5.351	0.597	15.4	−29.64	32.86
Touriga Franca (average of 3 years)	1.803	0.048 (0.020)	2.365	0.327	12.2	−19.11	19.69
Touriga Nacional (average of 12 years)	0.858	0.052 (0.006)	8.712	0.884	26.6	−57.81	51.76
Trajadura (average of 4 years)	5.632	0.790 (0.236)	3.346	0.359	15.8	−19.69	29.97
Trincadeira (average of 3 years)	1.087	0.020 (0.003)	6.648	0.583	12.8	−32.01	22.62
Uva Cão (average of 3 years)	4.834	1.022 (0.220)	4.653	0.873	20.9	−68.98	31.56
Verdelho (average of 3 years)	1.066	0.120 (0.024)	5.018	0.846	32.4	−69.55	56.16
Vinhão (average of 5 years)	5.308	0.758 (0.115)	6.585	0.651	16.4	−37.58	35.48
Viosinho (average of 8 years)	2.298	0.339 (0.042)	8.115	0.819	25.4	−63.00	44.59
Vital (average of 3 years)	1.875	0.334 (0.042)	7.956	0.748	30.8	−62.04	69.16

The residual likelihood ratio test for the genotypic variance component was performed ($H_{0}:\sigma_{g}^{2}=0\;\mathrm{vs}.\;H_{1}:\sigma_{g}^{2}>0$). For all studied cases, *p*-value < 0.05.

## Data Availability

Not applicable.
